# Cold-induced vasodilation comparison between Bangladeshi and Japanese natives

**DOI:** 10.1186/s40101-016-0095-5

**Published:** 2016-05-03

**Authors:** Aklima Khatun, Sakura Ashikaga, Hisaho Nagano, Md Abdul Hasib, Akihiro Taimura

**Affiliations:** 1 Graduate school of Fisheries and Environmental Sciences, Nagasaki University, 1-14 Bunkyo-machi, Nagasaki, 852-8521 Japan; 2 Graduate School of Engineering, Nagasaki University, 1-14 Bunkyo-machi, Nagasaki, 852-8521 Japan

**Keywords:** Cold-induced vasodilation (CIVD), Finger skin temperature, Thermal sensation

## Abstract

**Background:**

The human thermoregulation system responds to changes in environmental temperature, so humans can self-adapt to a wide range of climates. People from tropical and temperate areas have different cold tolerance. This study compared the tolerance of Bangladeshi (tropical) and Japanese (temperate) people to local cold exposure on cold-induced vasodilation (CIVD).

**Methods:**

Eight Bangladeshi males (now residing in Japan) and 14 Japanese males (residing in Japan) participated in this study. All are sedentary, regular university students. The Bangladeshi subject’s duration of stay in Japan was 2.50 ± 2.52 years. The subject’s left hand middle finger was immersed in 5 °C water for 20 min to assess their CIVD response (the experiment was conducted in an artificial climate chamber controlled at 25 °C with 50 % RH).

**Results:**

Compared with the Bangladeshi (BD) group, the Japanese (JP) group displayed some differences. There were significant differences between the BD and JP groups in temperature before immersion (TBI), which were 33.04 ± 1.98 and 34.62 ± 0.94 °C, and time of temperature rise (TTR), which were 5.35 ± 0.82 and 3.72 ± 0.68 min, respectively. There was also a significant difference in the time of sensation rise (TSR) of 8.69 ± 6.49 and 3.26 ± 0.97 min between the BD and JP groups, respectively (*P* < 0.05). Moreover, the JP group showed a quick TTR after finishing immersion.

**Conclusions:**

The Japanese group (temperate) has a higher tolerance to local cold exposure than the Bangladeshi group (tropical) evaluated by the CIVD test.

## Background

Cold acclimation is the process leading to the development of cold tolerance. Adaptation to cold environments played an important role in the survival of Homo sapiens during the last ice age and variations with respect to cold adaptation are reflected in human phenotypes today [1]. When humans are exposed to cold environments, vasoconstriction occurs to regulate heat loss; however, the degree to which the thermal environment can be adjusted by vasoconstriction is small and thermogenesis is required to maintain optimal body temperature. During cold exposure, the body attempts to maintain a constant internal body temperature by increasing heat production and minimizing heat loss, both of which are mediated by activation of the sympathetic nervous system [2].

Cold-induced vasodilation (CIVD) can be defined as vasodilation of cold-exposed blood vessels, in particular the small arteries. It is an acute increase in peripheral blood flow observed during cold exposure whereby the blood vessel diameter increases due to local cold. This counterintuitive acute increase in local cutaneous blood flow develops during cold exposure resulting in increased local tissue temperature, which may protect the region from cold injury [3]. Generally, CIVD is observed by increased blood flow or skin surface temperature. Age, gender, altitude, diet, alcohol consumption, and mental stress are individual factors that affect CIVD responses [4].

Research has been conducted to determine why CIVD occurs in tropical natives. Tropical indigenes have less active responses of arteriovenous anastomoses in the fingers and weaker vasoconstrictions after the first CIVD response during finger cold immersion [5]. Ethnicity is a risk factor for cold injury [6]; the tropical indigenes (African) are more vulnerable to non-freezing cold injury compared with Caucasian individuals [7]. In Lee et al.’s study [5], subjects were from different tropical countries but not from Bangladesh. Each country differed in climate temperature.

Bangladesh has a tropical monsoon climate with a mild winter and hot, humid summer. The annual temperature is 25.43 °C [8] and relative humidity is 78.45 % [9]. As there are many local people living outside of Bangladesh, it is important to know such people’s local cold tolerance. To date, little research has been conducted on this topic in Bangladeshi natives. Nagasaki (Japan) is located in a temperate zone with an annual temperature of 16.7 °C and humidity of 72.5 % [10]. In this present study, we investigated differences in the tolerance of local cold exposure between Bangladeshi and Japanese people on CIVD responses during finger immersion in cold water.

## Methods

### Subjects

Eight Bangladeshi and 14 Japanese sedentary male students (22 in total) participated in this study. They were verbally informed of the aims, risks, and benefits of this investigation, and written informed consent was obtained from all subjects before the start of the experiments. Before the experiment, the physical conditions of all subjects were measured. The Ethics Committee of the Faculty of Environmental Sciences, Nagasaki University approved this study. The eight Bangladeshi male subjects (age, 29.37 ± 3.02 years; height, 169.92 ± 3.42 cm; weight, 69.87 ± 11.22 kg; %body fat, 28.85 ± 7.91 %; BMI, 24.17 ± 4.19), who were born and raised in a tropical country (Bangladesh) and now resident in a temperate area (Nagasaki, Japan), and the 14 Japanese male subjects (age, 21.57 ± 0.93 years; height, 172.7 ± 5.78 cm; weight, 59.21 ± 5.23 kg; body fat, 16.7 ± 3.93 %; BMI, 19.89 ± 1.98) were born, raised, and live in a temperate country (Japan). Subjects in both groups were all students of the same university with an almost identical lifestyle. The diet of the tropical subjects was somewhat different to that of the temperate subjects. As Muslims, the tropical subjects never drink alcohol or eat pork and are all university students. After coming to Japan, their lifestyle did not change much. The duration of stay in Japan of all Bangladeshi subjects was not the same. Their lengths of stay in the temperate area were 2.50 ± 2.52 years. Table 1 shows the physical characteristics of all subjects of the Bangladeshi (BD) and Japanese (JP) groups.

**Table 1:** Physical characteristics of Bangladeshi (BD) and Japanese (JP) groups

Group		Height (cm)	Age (year)	Weight (kg)	Body fat (%)	BMI
BD	Mean	169.92	29.37*	69.87*	28.82*	24.17*
	SD	3.42	3.02	11.22	7.91	4.19
JP	Mean	172.7	21.57	59.21	16.70	19.89
	SD	5.23	0.93	5.23	3.93	1.98

The Bangladeshi group had eight subjects and the Japanese group had 14 subjects. There were significant differences in age, weight, body fat, and BMI (body mass index) between the two groups, *P < 0.05. All data are shown as mean ± S.D.

### Experimental procedures

The experiment protocol was as follows: subjects rested on a chair in an artificial climate chamber for 10 min; after resting, they immersed their finger (left hand middle finger) in 5 °C water for 20 min. After cold immersion, the finger was removed from the water tank and subjects rested for recovery for 10 min. The temperature of the artificial climate chamber was controlled at 25 °C with 50 % relative humidity. If any subject felt pain or intolerance in their finger, they were able to withdraw from the experiment at any time. Alcohol intake and exercise were prohibited at least 24 h before the experiment. All experiments were conducted in September 2014.

### Measurements

**Fig. 1:**
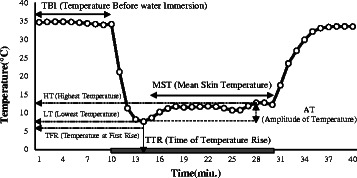
CIVD data of finger skin temperature during experiment

During the experiments (left hand middle finger immersion), the following CIVD data were collected: temperature before water immersion (TBI); temperature at first rise (TFR); highest temperature of 5–20 min after the start of immersion (HT); lowest temperature of 5–20 min after the start of immersion (LT); amplitude of temperature (AT), it is the difference between the LT and HT; time of temperature rise (TTR); mean skin temperature of 5–20 min after the start of immersion (MST); coefficient of variation of the temperature (CVT); and resistance index (RI). Figure 1 shows the sample data from that 40 min experiment. The black solid line indicates the subject’s finger skin temperature.

The following sensation data were also collected during the experiment time: sensation before water immersion (SBI); sensation at first rise after the start of immersion (SFR); time of sensation rise (TSR); highest sensation of 5–20 min after the start of immersion (HS); lowest sensation of 5–20 min after the start of immersion (LS); mean skin sensation of 5–20 min after the start of immersion (MSS); amplitude of sensation (AS), the difference between the LS and HS; and the coefficient of variation of the sensation (CVS).

Finger skin temperature was measured continuously using a thermistor sensor (PXK67, TECHNO SEVEN). The thermistor sensor on the left hand middle finger was attached just lateral of the nail bed. The skin temperature was sampled every 5 s. The thermal sensation was recorded every 2 s using the thermal sensation measurement software “takumiBG©” (esPRODUCTS). The thermal sensation scale was divided into eight stages from 0.0 (extremely cold) to 8.0 (extremely hot). Subjects recorded the thermal sensation by operating a mouse with their right hand (not immersed).

### Data analysis

All subject data were calculated and statistically presented as the mean ± standard deviation. An independent-sample *t* test was used to compare between the Bangladeshi and Japanese groups. Statistical analysis was performed using SPSS© software (version 21 for Mac). *P* < 0.05 was considered as a statistically significant difference.

## Results

**Fig. 2:**
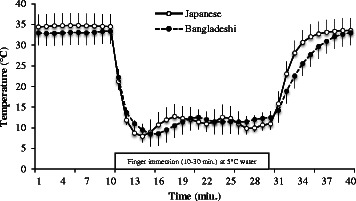
Average finger skin temperature of the Bangladeshi (BD) and Japanese (JP) groups. There were significant differences in temperature before water immersion (TBI) and time of temperature rise (TTR). The BD and JP group’s TBI was 33.04 ± 1.98 and 34.62 ± 0.94 °C; TTR was 5.35 ± 0.82 and 3.72 ± 0.68 min, respectively

Figure 2 shows the comparison of average finger skin temperature of the two groups. The dotted line indicates the BD group’s average finger skin temperature, and the solid line indicates the JP group’s average finger skin temperature. The experiment was conducted for 40 min: the first 10 min were rest, followed by 20 min of finger immersion, and the last 10 min were the rest and recovery stage of the experiment. After the start of finger immersion, the finger skin temperature decreased rapidly. Table 2 shows the CIVD index of the two groups. TBI (33.04 ± 1.98 and 34.62 ± 0.94 °C of BD and JP groups, respectively) and TTR (5.35 ± 0.82 and 3.72 ± 0.68 min of BD and JP groups, respectively) between two groups (*P* < 0.05). The summer season had more pronounced CIVD than any other time [4]. The CIVD wave numbers observed during the immersion period were 2.43 ± 0.67 and 2.78 ± 0.84 in the BD and JP groups, respectively. There were no significant differences in CIVD wave between the two groups.

**Table 2:** CIVD index of Bangladeshi (BD) and Japanese (JP) groups

Group		TBI (°C)	TFR (°C)	TTR (min)	MST (°C)	HT (°C)	LT (°C)	AT (°C)	CVT (%)	RI
BD	Mean	33.04	8.06	5.35*	11.38	14.66	7.12	7.34	17.11	11.87
	SD	1.98	3.67	0.82	2.96	3.87	1.77	2.75	5.92	1.72
JP	Mean	34.62*	7.51	3.72	11.43	13.97	8.04	5.93	14.43	11.14
	SD	0.94	1.67	0.68	1.98	2.74	1.70	2.44	5.65	1.35

* There were significant differences in TBI and TTR between two groups, *P* < 0.05

**Fig. 3:**
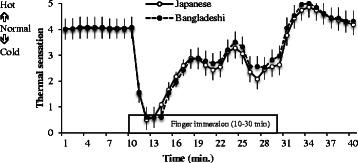
Average finger thermal sensation of the Bangladeshi (BD) and Japanese (JP) groups. There was a significant difference in TSR between the two groups. The BD and JP group’s time of sensation rise (TSR) was 8.69 ± 6.49 and 3.26 ± 0.97 min, respectively

Figure 3 shows a comparison of the average finger skin thermal sensation of the two groups. The dotted line indicates the Bangladeshi group’s average skin finger thermal sensation, and the solid line indicates the Japanese group’s average skin finger thermal sensation. Similar to the finger skin temperature, the same change occurred in the finger skin thermal sensation case. Table 3 shows the thermal sensation index of the two groups. There was a significant difference only in TSR between the two groups. The BD group’s TSR was 8.69 ± 6.49 min, which was higher than and JP (3.26 ± 0.97 min) groups.

**Table 3:** Thermal sensation index of Bangladeshi (BD) and Japanese (JP) groups

Group		SBI (°C)	SFR (°C)	TSR (min)	MSS (°C)	HS (°C)	LS (°C)	AS (°C)	CVS (%)
BD	Mean	4.00	1.15	8.69*	2.40	3.88	0.81	3.07	35.55
	SD	0.00	0.67	6.49	0.75	1.34	0.84	1.32	7.19
JP	Mean	4.03	0.96	3.26	2.64	3.94	0.99	2.95	35.56
	SD	0.11	0.62	0.97	0.63	0.71	0.94	1.20	17.14

* Significant different between two groups was TSR, *P* < 0.05

## Discussion

Humans have special heat and cold tolerance depending on their climate areas. Polar residents have superior CIVD response compared with tropical natives [11]. Tropical natives have superior tolerance to heat stress [12]. While heat acclimatization reflects the development of heat tolerance, it may weaken cold tolerance [5]. People from tropical (Bangladesh) areas and people from temperate (Japan) areas do not have the same heat/cold tolerance. Ethnicity was determined by self-classification, and there are some significant differences between ethnic groups [7]. The CIVD responses of tropical indigenes have received relatively little attention. It is generally accepted that humans have a tropical or subtropical origin [13], which posits that the temperature regulation of the human body is essentially adapted to subtropical, rather than colder climates. Cold adaptation could be reflected in the degree of attenuation of initial vasoconstriction in cold immersion fingers [14]. Due to the subtropical adaptation of the human body, it is reasonable to assume that the vasomotor activity of tropical indigenes to cold stimuli would not be developed sufficiently to protect from severe cold and remain fully functional. Tropical indigenes were less able to detect cutaneous warmth and had a wider inter threshold zone for cutaneous thermal sensation than temperate indigenes [15]. Finger CIVD responses were greater when body temperature was increased [16, 17] and dulled by decreases in body temperature [18, 19] and finally ceased when the temperature fell below baseline [20]. Tropical indigenes could be more vulnerable to cold injury of the peripheries in severe cold [5].

In the CIVD index, there were significant differences in TBI and TTR between the Bangladeshi and Japanese groups (*P* < 0.05). The JP group’s TBI was higher but the hunting reaction was influenced by core temperature, rather than skin temperature [4]. The results of Lee et al.’s study [5] showed that minimum temperature (*T*_min_), maximum temperature (*T*_max_), and mean temperature (*T*_mean_) were significantly different between the tropical and temperate groups during cold-water immersion. This same parameter in the present study showed that there were no significant differences in LT, HT, and MST between the groups. The JP group’s TTR was faster than that of the BD group. TTR (onset time) was significantly different between the groups (5.35 ± 0.82 and 3.72 ± 0.68 min). In the thermal sensation index, there was a significant difference in TSR between the groups (*P* < 0.05). The onset time (*t*_onset_) of the temperate group was double the length of that of the tropical group (8.6 ± 7.1 and 4.4 ± 1.8 min; *P* = 0.066) [5]. TTR (*t*_onset_) and TSR are important indices for evaluating local cold tolerance during cold-water immersion. The physical characteristics of subjects in the present study were significantly different in some aspects but in Lee et al.’s study [5], there was no significant difference except for age. The BD group’s %body fat was significantly higher; so LT, HT, and MST were not significantly lower than those in the JP group. Generally, any person with high body fat has high cold tolerance because body fat works as a protective layer against cold temperature. The human body is essentially adapted to a tropical rather than a colder climate. The tropical indigence vasomotor activity to cold stimuli would not be sufficiently developed to protect from severe cold and remain fully functional [5]; the CIVD reaction is more pronounced for people born in cold areas [4]. Although the present study only measured the finger cold exposure tolerance not total body cold exposure, %body fat had no effect on this local cold exposure. As Bangladesh is a tropical (mainly summer) country, the Bangladeshis had lower local cold tolerance than the Japanese (temperate natives). The present results of the length of stay, displaying no relation to the CIVD parameters for the tropical indigenes (Bangladeshi), appear to support the previous findings [5].

## Conclusions

The Japanese group (temperate) had higher tolerance to local cold exposure than the Bangladeshi group (tropical) evaluated by the CIVD test. After immersion, finger temperature recovery in the JP group was quicker than that in the BD group. Although Japanese subjects had low body fat, their local cold tolerance was higher than that of the Bangladeshi subjects. As the Japanese subjects grew up in temperate areas, they have acquired higher local cold tolerance than tropical indigenes.

## Abbreviations

AT: amplitude of temperature (HT-LT)
AS: amplitude of sensation (HS-LS)
CVS: coefficient of variation of the sensation
CVT: coefficient of variation of the temperature
HS: highest sensation (5~20 min)
HT: highest temperature (5~20 min)
LS: lowest sensation (5~20 min)
LT: lowest temperature (5~20 min)
MSS: mean skin sensation (5~20 min)
MST: mean skin temperature (5~20 min)
RI: resistance index
SBI: sensation before water immersion
SFR: sensation at first rise
TBI: temperature before water immersion
TFR: temperature at first rise
TSR: time of sensation rise
TTR: time of temperature rise
